# Evaluation of Residues of Amazonian Fruit Piquia (*Caryocar villosum)* as Sustainable Ingredient for Sunscreen and Cosmetic Formulations

**DOI:** 10.3390/antiox14020122

**Published:** 2025-01-21

**Authors:** Izadora de Souza, Gabriella C. P. Grimmelprez, Klenicy K. L. Yamaguchi, Johannes Schleusener, Silke B. Lohan, Martina C. Meinke, Lorena R. Gaspar

**Affiliations:** 1 School of Pharmaceutical Sciences of Ribeirão Preto, University of São Paulo, Av. do Café, s/n, Vila Monte Alegre, Ribeirao Preto 14040-900, SP, Brazil; 2 Department of Dermatology, Venereology and Allergology, Charité–Universitätsmedizin Berlin, Corporate Member of Freie Universität Berlin and Humboldt-Universität zu Berlin, Charitéplatz 1, 10117 Berlin, Germany; 3 Institute of Health and Biotechnology, Federal University of Amazonas, Coari 69460-000, AM, Brazil

**Keywords:** amazonian fruit residues, piquia shell extract, UVA and UVB protection potential, natural photostabilizer, cumulative radical production induced by VIS + NIR, electron paramagnetic resonance spectroscopy, confocal Raman microspectroscopy

## Abstract

Amazonian fruit residues like piquia shells are often discarded despite their antioxidant potential for sustainable cosmetic use. This study evaluated the photostability, phototoxicity, and photoprotection of hydroalcoholic piquia shell extract (PqSE) combined with UV filters in solutions and cosmetic formulations. PqSE formulations were photostable, even stabilizing photounstable UV filters. Phototoxicity tests (OECD TG 432) showed no phototoxic potential (MPE < 0.15) and reduction in the phototoxic potential of UV filters, while ocular irritation potential via HET-CAM assay indicated no irritant effects. The extract combined with UV filters enhanced protection against UVA-induced reactive oxygen species (ROS) production, achieving 60.9% effectiveness, outperforming commercial photostabilizers. Against UVB radiation, it showed cellular viability above 80%, comparable to benzophenone-3. PqSE formulations exhibited a radical protection factor (RPF) nine times higher than controls and reduced radical production by 64% after visible/near-infrared (VIS/NIR) irradiation on porcine skin, compared to 38% for controls. Confocal Raman microspectroscopy showed penetration depths below 12 µm for all time points. This study highlights the potential of reusing fruit residues like PqSE as sustainable, effective ingredients in sunscreen formulations, offering enhanced photoprotection and reduced environmental waste.

## 1. Introduction

Sunlight is composed of infrared (IR) radiation, visible light (VIS), and ultraviolet (UV) UVA, UVB, and UVC radiation. Part of this radiation is absorbed by the ozone layer; however, part of it reaches the Earth and can cause damage to our skin, such as sunburns, skin cancer, erythema, and photoaging [1]. The use of sunscreens is an important strategy for protecting the skin against damage caused by sunlight. Sunscreen formulations are composed mainly of UV filter molecules, which are able to protect the skin by absorbing or reflecting radiation. A suitable sunscreen formulation should combine UV filters aiming to have broad-band light absorption, including UVA, UVB, and VIS/IR. It is also important that UV filters present chemical photostability to resist the radiation to which they will be exposed.

The UV filter combination of avobenzone (AVO), a broad-spectrum and UVA-I filter (340–400 nm), and octyl methoxycinnamate (OMC), a UVA-II (320–340 nm) and UVB (290–320 nm) filter is well known for being photounstable. AVO may affect the photostability of other UV filters, and for instance, the photodegradation of the UVB filter OMC is accelerated in the presence of avobenzone by [2 + 2]-cycloaddition [2,3].

Avobenzone has a keto-enol equilibrium that can absorb strongly in the UVA region without irradiation due to the predominance of the enol form. After exposure to irradiation, this equilibrium is displaced for the photoreactive *β*-diketo isomer formation. In this context, a loss of UV-protecting capability is associated with the decrease in the enol UVA band centered at 360 nm and the concomitant increase in the *β*-diketone absorption at 260–280 nm [4,5]. This UV-B filter may be photounstable because OMC undergoes photoisomerization from the trans-isomer to the cis-isomer after exposure to sunlight, losing its photoprotective effect [6]. Several strategies have already been proposed to improve their photostability, these include their combination with other UV filters, such as octocrylene, photostabilizing molecules, and antioxidants, incorporating them into lipid microparticles [4,7,8,9].

Studies indicate that the addition of antioxidants to sunscreens can protect against the production of reactive oxygen species (ROS) by UV irradiation, enhancing the effects of UV filters, and increase overall UV absorption [10,11]. In addition, the cosmetic industry has found that natural-origin compounds are being chosen because consumers are more concerned and informed about the importance of sustainability and environmental responsibility [12].

Thus, several safety and efficacy studies have been performed with the aim of finding new natural raw materials to be used in such formulations. Piquia (*Caryocar villosum*) is a common fruit in the Amazon Rainforest used mainly for food purposes. However, studies indicate that the extract of piquia pulp presents not only relevant reactive nitrogen species and ROS protection but may also have antigenotoxic properties [13,14].

According to the Food and Agriculture Organization (FAO) of the United Nations, Brazil is the third largest fruit producer in the world, having produced about 59 million tons in 2020. About 2.5% of the production is exported, and the 97.5% that is available for internal consumption can generate up to 40% of residues [15,16]. The reuse of fruit residues has been gaining relevance and is being extensively studied, constituting an important option to minimize the possible environmental impacts arising from the inadequate disposal of residues produced by fruits and fruit derivative industries [17,18,19].

Some studies found in the literature involving residues of Amazonian fruits, which are usually discarded, were carried out in order to add value to these residues and to promote their sustainable use. Among these studied fruits, piquia shells (*Caryocar villosum*) were highlighted due to their antioxidant activity tested by 2,2′-azino-bis(3-ethylbenzothiazoline-6-sulfonic acid (ABHT) measurements [20,21]. The piquia shells represent about 65% of the weight of the fruit and it is a solid waste from piquia processing that is normally discarded. The shells are rich in fibers and phenolic compounds, such as ellagic and gallic acids [22,23]. With a relevant antioxidant potential, the use of piquia shell extract in a sunscreen formulation would be interesting.

Thus, this study proposes the combination of piquia shell extract with UV filters AVO and OMC in a formulation to evaluate its toxicity, photosafety, and photoprotection against UVA, UVB, and VIS/IR radiation. This study regards some of the sustainable development goals (SDG) from the United Nations since it is a contribution to sustainable development in social, economic, and environmental fields. It is aligned with SDG number 2 in terms of sustainable agriculture since the reuse of shells from the Amazonian fruit piquia, which would be discarded, may have added value to this material and could help small producers. It is also aligned with SDG number 14, which suggests the prevention and reduction in marine pollution, in which the use of fruit residues in photoprotective formulations can reduce the UV filters in formulations and, consequently, reduce the contamination of corals by UV filters [24].

## 2. Materials and Methods

### 2.1. Materials

Ethanol, isopropanol, glycerin, and propylene glycol were purchased from Labsynth (Diadema, Brazil). Glacial acetic acid was purchased from J.T. Baker (Phillippsburg, NJ, USA). Alkyl benzoate C12-15 (Crodamol^TM^ AB) and isopropyl myristate (Crodamol ^TM^ IPM) were obtained from Croda (Snaith, UK). Cetearyl alcohol and cetearyl glucoside (Montanov 68) and hydroxyethyl acrylate/sodium acryloyldimethyltaurate copolymer and squalane and polysorbate 60 (Simulgel_ NS) were obtained from Seppic (Paris, France). Norfloxacin, quercetin, glutamine, antibiotic mixture (penicillin, streptomycin, and amphotericin B), and dimethyl sulfoxide (DMSO) were purchased from Sigma-Aldrich (St. Louis, MO, USA). The fetal bovine serum and Dulbecco’s modified Eagle medium (DMEM) were purchased from Gibco (Carlsbad, CA, USA). The calf serum was purchased from Hyclone (Logan, UT, USA). Neutral red from Merck (Darmstadt, Germany), butylated hydroxytoluene (BHT) from Mapric (São Paulo, Brazil), and cyclomethicone from GE Silicones (Wilton, CT, USA).

Nitroxide PCA (3-carboxy-2,2,5,5-tetramethyl-1-pyrrolidinyloxy) and the radical DPPH (1,1-diphenyl-2-picrylhydrazyl) were both purchased from Sigma-Aldrich (Steinheim, Germany) and were used for electron paramagnetic resonance (EPR) investigations. Ethanol (UVASOL, Merck, Germany) was utilized for the RPF determination.

The UV filters were butyl methoxydibenzoylmethane (avobenzone; Eusolex 9020) from Merck (Darmstadt, Germany), octyl methoxycinnamate (Octinoxate, Uvinul_ MC80) from BASF (Ludwigshafen am Rhein, Germany), and (2-Hydroxy-4-methoxyphenyl)-phenylmethanone (benzophenone-3; Neo Heliopan BB) from Symrise (Holzminden, Germany). The photostabilizer ethylhexyl methoxycrylene (Solastay S1) was obtained from Hallstar Company (Chicago, IL, USA).

Samples of the piquia shells were kindly provided by Cooperativa Agrícola Coari Itapeua (CAECI) (Coari, Amazonas, Brazil). The voucher specimen of piquia was deposited at the Carpoteca of Federal Institute of Amazonas—IFAM/Manaus under number C1395 and also registered at National System for Management of Genetic Heritage (SISGEN) under number A3B96EB. Piquia shells were washed, dried, and milled. The hydroalcoholic extract was obtained by maceration method for 72 h, at room temperature, using ethanol/water (7:3). The ratio of weight of raw material/volume of hydroalcoholic mixture was 1:10, respectively. The extract was then lyophilized and stored at −20 °C. The reconstitution of the lyophilized material was performed using a solvent mixture of ethanol and water (7:3).

UV filters and piquia shells hydroalcoholic extract combinations were tested in solution in PBS and were also added in cosmetic formulations as described in the following items.

### 2.2. Preparation of Sunscreen Formulations

The experimental formulations were developed using a self-emulsifying wax (cetearyl alcohol, cetearyl glucoside) (Montanov 68^®^) combined with a liquid polymer and surfactant blend (hydroxyethyl acrylate, sodium acryloyldimethyl taurate copolymer, squalane and polysorbate 60) (Simulgel NS^®^), as outlined in Table 1 [2]. The formulation FAO contained additionally a combination of two UV filters, 8% OMC and 4% AVO. These were supplemented or not with 5% of ethylhexyl methoxycrylene (EHMCR) FAOE and 1% or 5% of piquia shell hydroalcoholic extract (PqSE) FAOPq (Table 1).

### 2.3. Photostability Study for Formulations

The photostability study was performed for formulations FAO, FAOE, and FAOPq. Each formulation was spread onto an area of 10 cm^2^ (approximately 4 mg/cm^2^) of a glass plate and then left to dry for 15 min before exposure to a UVA dose of 27.5 J/cm^2^. This dose is equivalent to 66 min of midday sunlight exposure (6.94 mW/cm^2^) on a typical sunny September day in Ribeirao Preto, Brazil, located at latitude 21°10′39″ S and longitude 47°48′37″ W. [25,26]. One glass plate was irradiated, and the other was kept in a dark place [11,27]. Samples exposed or not to UVA were resuspended in isopropanol, and the dried film was dissolved in the ultrasound bath. Samples were diluted (1:10, *v*/*v*), and the ratio of mean UVA (320–400 nm)/mean UVB (280–320 nm) absorbances was calculated as [26,28].

### 2.4. Toxicity Evaluation

#### 2.4.1. Phototoxicity Assay (3T3 PT NRU)

3T3 Neutral red uptake phototoxicity (3T3 NRU PT) test was performed according to OECD 432 guideline [29] (OECD, 2019) using 3T3 BALB/c fibroblasts cells from the Cell Bank of Rio de Janeiro (Rio de Janeiro, Brazil). Briefly, fibroblasts were cultured in DMEM medium supplemented with calf serum, L-glutamine, and antibiotic mixture and incubated at 37 °C. The DMSO stock solutions of combinations CAO: AVO and OMC; CAOE: AVO, OMC and EHMCR; CAOPq: AVO, OMC, and PqSE were used at the same proportions as used in formulations under study (4:8:5) and at proportion (5:7:5) considered more phototoxic [2]. These two proportions (4:8 and 5:7) of the UV filter combination (CAO: AVO and OMC) were evaluated in order to choose one with phototoxic potential. Then, it was possible to evaluate if the well-known photostabilizer EHMCR and the studied extract PqSE would be able to reduce the phototoxicity of the UV filters.

The initial concentrations of the substances under study for the proportion 4:8:5 were 50 µg/mL for AVO, 100 µg/mL for OMC, 62.5 µg/mL for EHMCR, 62.5 µg/mL for PqSE and the initial concentrations of the substances under study for the phototoxic proportion (5:7:5) were 71.4 µg/mL for AVO, 100 µg/mL for OMC, 71.4 µg/mL for EHMCR, 71.4 µg/mL for PqSE. These preparations were also diluted in phosphate-buffered saline (PBS, pH 7.2) to generate samples with 8 different concentrations in a geometric progression (constant factor = 1.47). The highest final concentration of DMSO was 1%.

Fibroblasts were seeded at a density of 10^4^/well. After 24 h of incubation, they were treated with eight different concentrations of the combinations in sextuplicate. Following this, the cells were incubated for 1 h (5% CO_2_; 37 °C) and then irradiated with UVA light at a dose of 9 J/cm^2^. After irradiation, the plates were incubated for 18–22 h (5% CO_2_, 37 °C). Cell viability was measured by neutral red uptake (NRU) assay according to OECD 432 guideline [29] (OECD, 2019), and the absorbance was measured at 540 nm (microplate reader Synergy™ 2#, Biotek, Winooski, VT, USA). To evaluate the concentration response, the Phototox Version 2.0 software (ZEBET, Berlin, Germany) was used. According to the test prediction model, which is based on mean photo effect (MPE), a test substance with values below 0.1 predicts “non-phototoxic”; values between 0.1 and 0.15 are predicted as “probably phototoxic” and values greater than 0.15 predict “phototoxic” [29,30,31].

#### 2.4.2. Ocular Irritation Potential

The HET-CAM (Hen’s Egg Test—chorioallantoic membrane) is a toxicological assessment to verify ocular irritation potential. It was performed on chorioallantoic membrane, which is highly vascularized, in embryonated chicken eggs. White Leghorn chicken eggs were incubated, and on the 10th day of incubation, 0.1 g of formulations of FAO, FAOE, or FAOPq were applied. SDS solution (1%, *w*/*v*) was used as a positive control, and 0.9% NaCl solution (*w*/*v*) was used as a negative control. Phenomena were observed and filmed for up to 5 min. According to Luepke [32], the effects of hyperemia, coagulation, and hemorrhage can be observed, and it is attributed one score for each observed effect according to the time of appearance, which is used to calculate the irritation score obtained by the sum of individual scores. This score (S) is necessary to classify the tested substance as practically non-irritating (S ≤ 1), slightly irritating (1 < S ≤ 5), moderately irritating (5 < S ≤ 9), and irritating (S > 9). The test was performed in quadruplicate. [32]

### 2.5. Efficacy Evaluation

#### 2.5.1. UVB Photoprotection Assay

Keratinocytes HaCaT (immortalized human keratinocytes), obtained from the Rio de Janeiro Cell Bank (Rio de Janeiro, Brazil), were seeded in 96-well microplates at a density of 10^5^ cells/well and incubated for 24 h (5% CO_2_; 37 °C). To evaluate the UVB photoprotective effect, DMSO stock solutions of combinations CAOE: AVO, OMC, and EHMCR and CAOPq: AVO, OMC, and PqSE were used at the same proportions as used in formulations under study (4:8:5). Previous cytotoxicity studies were performed to ensure a safe range of concentration. The initial concentrations of the substances under study for the proportion 4:8:5 were 23.14 µg/mL for AVO, 46.28 µg/mL for OMC, 28.92 µg/mL for EHMCR, 28.92 µg/mL for PqSE. These preparations were also diluted in phosphate-buffered saline (PBS, pH 7.2) to generate samples with 3 different concentrations in a geometric progression (constant factor = 1.47). The highest final concentration of DMSO was 1%. Benzophenone-3 (BZP) is a UV filter with high absorption at UVB and UVA-II range, and it was used as an organic UV filter control at a concentration of 100 µg/mL. The plates were kept in the dark or irradiated with a UVB cytotoxic dose (UVB lamp Broadband TL 40 W/12 RS#, Philips, The Netherlands) of 300 mJ/cm^2^ using an irradiance of 0.31 mW/cm^2^ for 16 min. The untreated irradiated cells and untreated non-irradiated cells were used as controls. After irradiation, the cells were washed, the medium was refilled, and the plates were incubated for 24 h. Cells were washed with PBS and incubated with culture medium containing neutral red as described in Section 2.4.1, and the absorbance was measured at 540 nm. Cell viability was calculated considering non-irradiated (UV-) and non-treated control as 100%. The assays were performed in triplicate in three independent experiments [33].

#### 2.5.2. Photoprotection Against UVA-Induced ROS Production

The 2,7-dichlorodihydrofluorescein-diacetate probe (DCFH_2_-DA) is frequently used in the evaluation of ROS, such as hydroxyl radical, hydrogen peroxide, and carbonate anion.

For the cytotoxicity test, 1 × 10^5^ cells (3T3 murine fibroblasts) were seeded in a 96-well plate and incubated for 24 h (5% CO_2_; 37 °C). The DMSO stock solutions of combinations CAO: AVO and OMC; CAOE: AVO, OMC and EHMCR and CAOPq: AVO, OMC and PqSE were used at the same proportions as used in formulations under study (4:8:5). The concentrations of the substances under study were C1 (37.02 µg/mL for AVO, 74.04 µg/mL for OMC and 46.4 µg/mL for EHMCR or extract), C2 (54.42 µg/mL for AVO, 108.84 for µg/mL OMC and 68.1 µg/mL for EHMCR or extract), and C3 (80 µg/mL for AVO, 160 µg/mL for OMC and 100 µg/mL for EHMCR or extract) were added to the 96-well plate and incubated for 1 h. Then, the tested samples were replaced with culture medium, and the plates were incubated for 18–22 h. Cells were washed with PBS and incubated with culture medium containing neutral red as described in Section 2.4.1, and the absorbance was measured at 540 nm. Sodium dodecyl sulfate (SDS) at 100 µg/mL was used as a cytotoxic control.

To perform photoprotection against UVA-induced ROS production assay using DCFH_2_-DA probe, 3T3 fibroblasts were used under the same conditions (density and exposure time) of the cytotoxicity test that was described above. Then, fibroblasts were seeded in 96-well plates for 24 h. After this, non-cytotoxic concentrations of the previous study were incubated for 1 h. Cells were washed with PBS, and 100 µL of the DCFH_2_-DA probe (10 µM) were added to the wells, followed by incubation for 30 min. Then, the plates were washed with PBS and exposed to a dose of UVA radiation (solar simulator Dr. Hönle type SOL-500, Munich, Germany) of 4 J/cm^2^. This dose is sufficient to generate ROS and not be cytotoxic to cells. Fluorescence was measured in a microplate reader at wavelengths of 488 nm (excitation) and 528 nm (emission). The assays were performed in triplicate in three independent experiments. The fluorescence of untreated irradiated cells was considered to be 100% in order to calculate the relative percentage of samples. Norfloxacin was used as a pro-oxidant control and quercetin was used as an antioxidant control.

#### 2.5.3. Evaluation of Radical Protection Factor (RPF)

Formulations F, FAO, FAOE, and FAOPq (with 1% and 5% of PqSE) were evaluated for their antioxidant capacity by EPR spectroscopy using the test radical 2,2-Diphenyl-1-picrylhydrazyl (DPPH, Sigma-Aldrich, Steinheim, Germany). A total of 250 mg of formulations were diluted in 10 mL of ethanol. A total of 400 µL of this solution was added into a tube containing 400 µL of 1 mM DPPH. These samples were kept in the dark, at room temperature, and under constant swiveling. RPF measurements were performed using an X-Band spectrometer (MS5000, Magnettech GmbH, Freiberg Instruments GmbH, Freiberg, Germany). Analyses were performed at 0, 19, 20, 21, and 22 h. The number of reduced DPPH radicals represents the antioxidant activity per 1 mg of substance [34] and is expressed as RPF = N × 10^14^ radicals/mg. N is calculated to Equation (1), where RC is the concentration of a test radical in (radicals/mL), RF is a reduction factor as signal intensity ratio of untreated test radical and test radical after treatment (normalized to the signal of the untreated test) and PI is the product input representing the amount of the substance/product measured (mg/mL) [34].

(1) $$RPF=\frac{RC\times RF}{PI}$$

#### 2.5.4. Quantification of Cumulative Radical Production Induced by VIS + NIR Irradiation on Porcine Ear Skin by EPR Spectroscopy

Cumulative radical production on porcine ear skin ex vivo during VIS/NIR irradiation was evaluated with an X-Band EPR spectrometer with a TMHS resonator (Elexys E 500, Bruker Bio Spin GmbH, Karlsruhe, Germany)) using 3-(carboxyl)-2,2,5,5-tetramethyl-1-pyrrolidinyloxy (PCA) (Sigma-Aldrich, St. Louis, MO, USA) as spin probe and established magnetic parameters [35,36]. The formulations F, FAOE, and FAOPq (PqSE 1%) were tested.

For the experiments, intact porcine ear skin sourced from a local butcher was utilized. The experiments were approved by the Veterinary Office Dahme-Spreewald, in accordance with Section 3, Article 17, paragraph 1, of Regulation (EC) No 1069/2009 of the European Parliament and of the Council of 21 October 2009, which establishes health rules for animal by-products not intended for human consumption.

Skin measurements were conducted 48 h post-slaughter. The porcine ears were cleaned with cold water, and the hair was carefully shaved without damaging the skin. The skin was stored at 4 °C in the dark until use. By using a dermatome (Aesculap-Werke, Tuttlingen, Germany), split skin samples of 300 μm thickness were prepared. The skin was maintained in a wet environment to prevent drying until use. Twenty µL/cm^2^ of formulation was applied on the skin surface and was evenly distributed on the skin surface for 2 min using a massage device (Rehaforum Medical GmbH, Elmshorn, Germany) followed by 30 min incubation time at room temperature. Afterward, 8 mm skin biopsies were taken and placed in a plate containing two filter papers of 12 mm in diameter soaked with 150 μL of a 1.0 mM PCA solution in PBS. After 5 min incubation time at 32 °C, a 4 mm (Ø) skin biopsy was taken, placed into a tissue cell, and measured in the EPR device. Measurements were performed for 15 min without irradiation and 15 min using a VIS-NIR optical fiber (LOT Oriel GmbH & Co. KG, Darmstadt, Germany) along with a sun simulator filter and a >400 nm 400FH90-50S filter. The intensity of each spectrum was measured both before and after each measurement using a power meter (843-R, Newport Corporation, Irvine, CA, USA). In total, six independent trials were conducted.

#### 2.5.5. Confocal Raman Microspectroscopy (CRM)

The penetration depths of formulations F, FAOE, and FAOPq (PqSE 1%) were determined on porcine ear skin using confocal Raman microspectroscopy (CRM). For the experimental procedure, six porcine ears were cleaned with cold water, and the hair on the surface was carefully shaved. Subsequently, the skin was stored at 4 °C until use.

An amount of 20 µL/cm^2^ was applied to porcine ear skin, and measurements were performed after 30 min of application on the skin (room temperature 21 °C). The excess cream was removed from the skin surface, and CRM measurements were taken at seven different points at the excised skin sample using a confocal Raman microscope (Model 3510 SCA, River Diagnostics B.V., Rotterdam, The Netherlands) [37,38]. Excitation at 785 nm (20 mW, 1.1 J/cm^2^ on the skin surface) was used for analysis in the fingerprint region (400–2000 cm^−1^). Raman fingerprint spectra were obtained from the skin surface to a depth of 40 µm in 2 µm increments. In this case, Raman spectra were obtained within the epidermis. Skin areas without furrows and hair were chosen to exclude their influence on penetration.

To determine the average depth of penetration of the formulations at each measurement point, the spectra of treated substances and untreated skin were compared by an adapted multiple least squares regression method using Skin Tools 2.0 Software (River Diagnostics). With this, it was possible to calculate depth-dependent scores of each individual skin compound (such as cholesterol, ceramide, keratin, urea, and water), and acquired spectra from the applied formulations, mirroring the spectra obtained by their known model spectra and thus minimizing the residual fit error. These depth-dependent scores provide information on the semi-quantitative concentration of each individual compound.

### 2.6. Statistical Analysis

For statistical analysis, Minitab 19 software (State College, PA, USA) and IBM SPSS^®^ Statistics Version 27.0 (IBM; Armonk, New York, NY, USA) were used, with *p* ≤ 0.05 set as statistically significant. ANOVA test followed by Tukey’s multiple comparison test) were used to detect differences between untreated and treated skin samples.

Evaluation of the EPR data of excised porcine ear skin was based on the Mann–Whitney *U* test for non-related samples. The generalized estimating equations (GEE) were used to compare curve progressions over time. All data are given as mean value (MW) ± standard error of mean (SEM). A level of *p* ≤ 0.05 was defined to be significant.

## 3. Results

### 3.1. Photostability of Formulations

**Figure 1:**
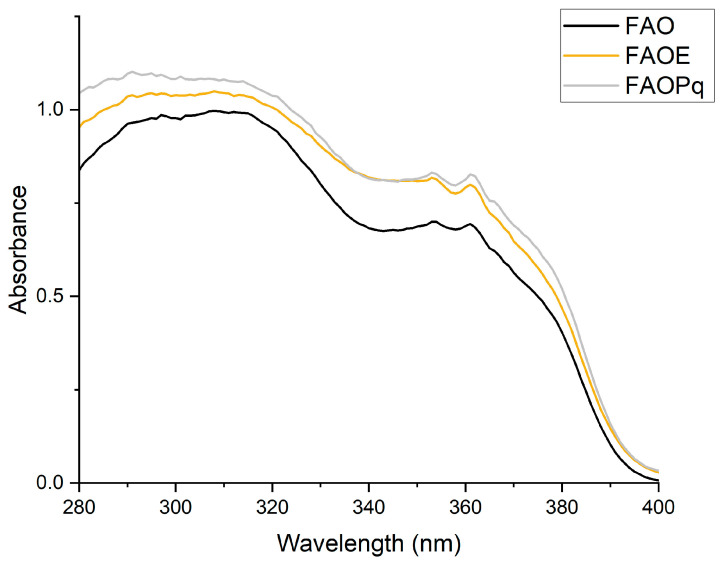
Absorption spectra in the UV region (280–400 nm) of formulations FAO (AVO + OMC), FAOE (AVO + OMC + EHMCR), and FAOPq (AVO + OMC+ 5% of PqSE). The results are expressed as mean absorbance (*n* = 3).

Absorption spectra of formulations FAO (AVO + OMC), FAOE (AVO + OMC + EHMCR), and FAOPq (AVO + OMC + PqSE) are shown in Figure 1. Formulation FAO showed an intense absorption in the UVA and UVB range: AVO at UVA-I (340–400 nm) and OMC at UVA-II (320–340 nm) and UVB (290–320 nm). When EHMCR and PqSE were combined in formulations FAOE and FAOPq, respectively, there was an increase in UVA-II absorption when compared to FAO.

**Figure 2:**
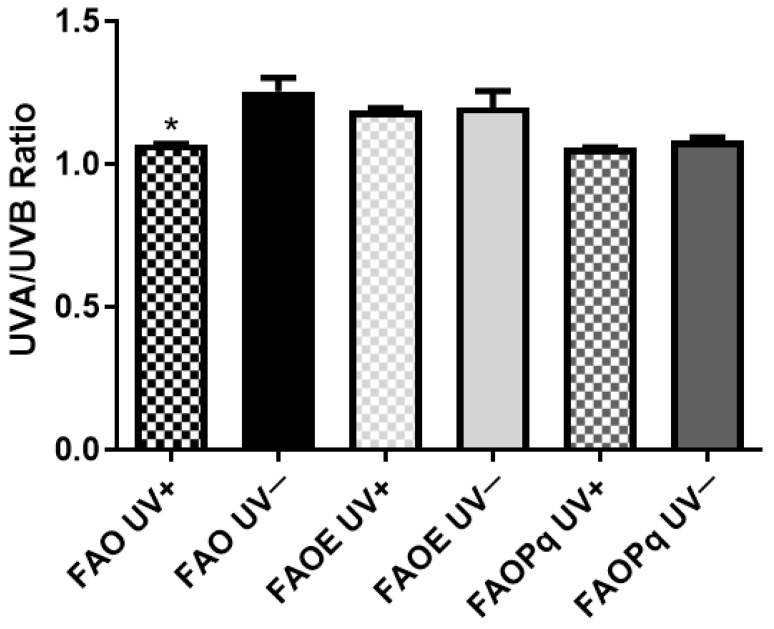
UVA/UVB ratios of irradiated (textured bars) and non-irradiated (plain bars) formulations FAO (AVO + OMC), FAOE (AVO + OMC + EHMCR), and FAOPq (AVO + OMC+ 5% of PqSE). UV+: irradiated formulation; UV-: non-irradiated formulation. Results are expressed as mean ± SEM (*n* = 3). Statistical analysis was performed using one-way ANOVA followed by Tukey’s test (*p* < 0.05). * Statistically different from formulation FAO UV−.

The UVA/UVB ratio of the non-irradiated pair was compared with its irradiated pair (Figure 2). FAO presented a high UVA/UVB ratio (1.254) but was considered photounstable since there was a statistically significant reduction in the UVA/UVB ratio after irradiation (1.068) (*p* < 0.05), probably due to AVO photodegradation. In addition, formulations FAOE and FAOPq exhibited better photostability than FAO and were considered photostable. No significant difference in photostability could be found for the UVA/UVB ratio of non-irradiated and irradiated formulations (*p* > 0.05).

### 3.2. Phototoxicity

The positive control norfloxacin was classified as phototoxic (MPE: 0.501; 0.434) and was within the MPE range recommended by the OECD Test Guideline 432 (0.340 to 0.900) (OECD, 2019) (Table 2).

Only one of the studied CAO combinations (AVO and OMC), proportion 5:7, presented phototoxic potential (MPE: 0.233 and 0.269) and was chosen to test EHMCR and PqSE potential. The other one (4:8) did not present any phototoxic potential (MPE: 0.035 and 0.036).

Both commercially available photostabilizer (EHMCR) and studied extract (PqSE) reduced CAO (5:7) phototoxic potential and were considered non-phototoxic (MPE CAOE*: −0.046; 0.007) and (MPE CAOPq*: 0.005; 0.073).

### 3.3. HET-CAM

**Figure 3:**
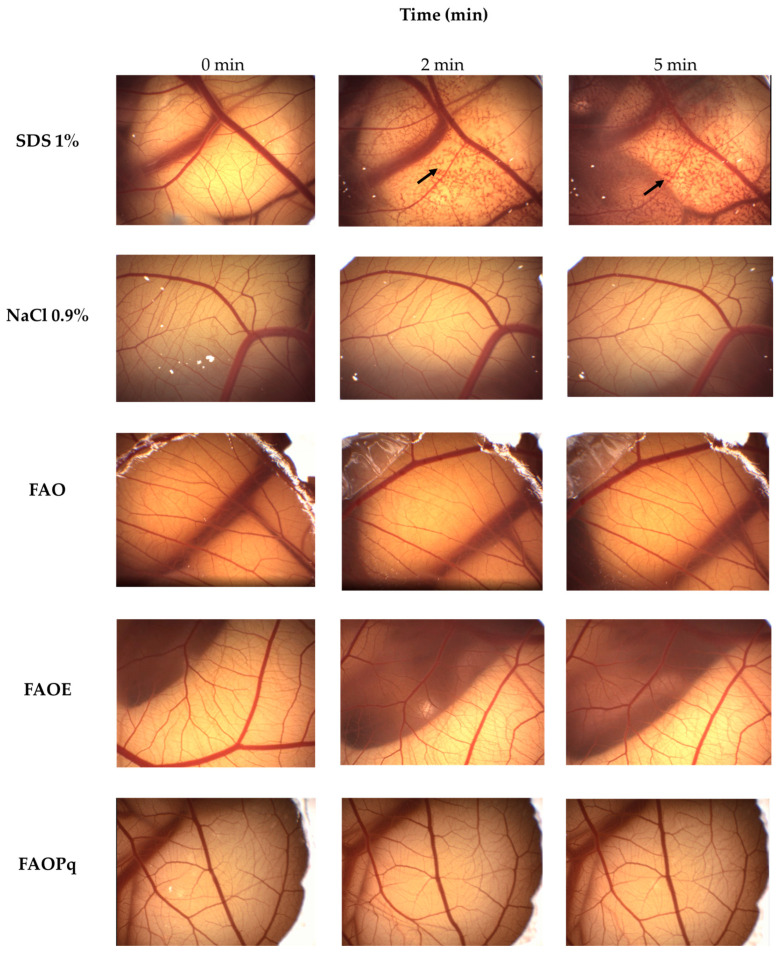
Evaluation of the irritant potential of formulations FAO (AVO + OMC), FAOE (AVO + OMC+ EHMCR), and FAOPq (AVO + OMC + 5% of PqSE) by the HET-CAM assay. The vascular effects were monitored for 5 min after exposure to each formulation. The sodium lauryl sulfate (SDS; 1%) and sodium chloride (NaCl; 0.9%) solutions were used as positive and negative controls, respectively. **↑**: indicates hemorrhage.

The positive control for eye irritation (SDS 1%) caused hemorrhage, with an irritation score equal to 12.0 ± 0, being classified as a severe irritant (Table 3, Figure 3). Negative control NaCl 0.9% did not present any damage to the chorioallantoic membrane, being classified as non-irritant (Score = 0 ± 0). Also, formulations of FAO, FAOE, and FAOPq had scores equal to 0 ± 0, with no effect observed on the chorioallantoic membrane during 5 min, being classified as non-irritant (Table 3, Figure 3).

### 3.4. UVB Photoprotection Assay

**Figure 4:**
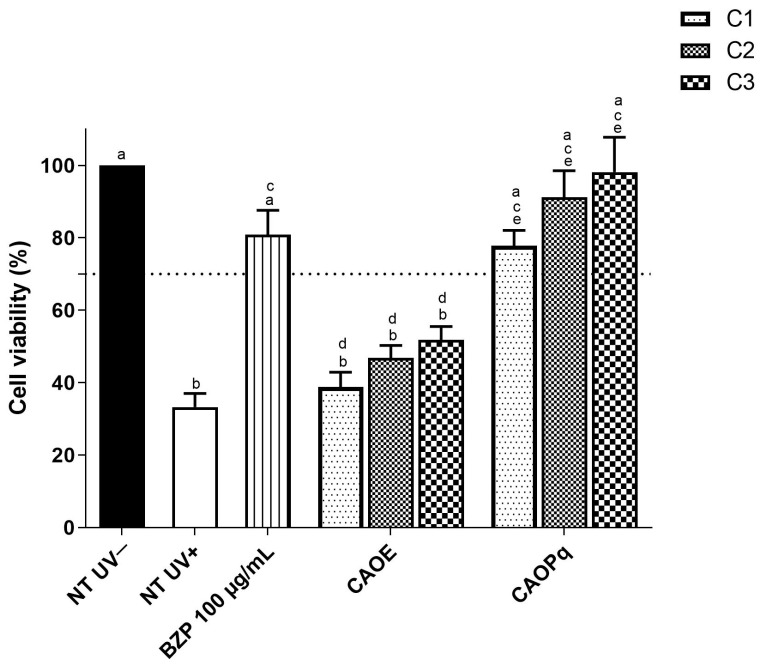
HaCaT cell viability followed by UVB irradiation (300 mJ/cm^2^). Combinations tested in the 4:8:5 proportion: CAOE (AVO, OMC, and EHMCR); CAOPq (AVO, OMC, and 5% of PqSE). Positive control: Benzophenone-3 (BZP; 100 μg/mL). NT—UV = non-treated and non-irradiated cells; NT + UV, non-treated and irradiated cells. AVO: avobenzone; OMC: octyl methoxycinnamate; EHMCR: ethylhexyl methoxycrylene; PqSE: piquia shell hydroalcoholic extract. Concentrations C1 (AVO = 10.71 µg/mL; OMC = 21.42 µg/mL; EHMCR or PqSE = 13.38 µg/mL), C2 (AVO = 15.74 µg/mL; OMC = 31.48 µg/mL; 19.68 µg/mL EHMCR or PqSE), and C3 (AVO = 23.14 µg/mL; OMC = 46.28 µg/mL; EHMCR or PqSE = 28.92 µg/mL). The results are expressed as mean ± standard errors of the mean of three independent experiments (*n* = 3). Bars with identical letters indicate groups with no significant difference (*p* > 0.05) (a ≠ b ≠ c ≠ d ≠ e).

To evaluate protection against UVB irradiation on HaCaT keratinocytes, the combinations CAOE and CAOPq were tested (Figure 4). A ratio of 4:8:5 (AVO OMC+ EHMCR/PqSE, respectively), considered non-phototoxic, was used.

In the UVB photoprotection assay, the viability of non-treated irradiated (NT UV+) cells decreased to 33% when compared to the non-treated and non-irradiated (NT UV−) cells (*p* < 0.05). Benzophenone-3 (BZP) was used as a control and enhanced cell viability to 80% when compared to NT UV+ (*p* < 0.05) and was considered not significantly different from (NT UV−) (*p* > 0.05).

CAOPq at concentrations C1, C2, and C3 significantly enhanced cell viability when compared to the non-treated irradiated cell group (NT + UV) (*p* < 0.05) (78%, 91%, and 98%, respectively) and also had a protective effect against UVB on HaCaT cells not significantly different to that of benzophenone-3.

On the other hand, CAOE, when used at the same concentration (C1, C2, and C3), did not enhance cell viability when compared to the non-treated irradiated cell group (NT + UV) (*p* > 0.05) (39%, 47%, and 52%, respectively) and the UVB protection was statistically lower than that of benzophenone-3. CAOPq offered a statistically higher protective effect against UVB on HaCaT cells than CAOE in all studied concentrations (*p* < 0.05).

### 3.5. Photoprotection Against UVA-Induced ROS Production

To ensure a safe concentration range, the cell viability was determined. A combination of CAO and CAOE was not considered to be cytotoxic at any of the concentrations tested. However, for CAOPq, concentrations 68.1 and 100 µg/mL for PqSE, respectively, were considered cytotoxic, with a decrease in cell viability of 53% and 96%, respectively. Consequently, only concentration C1 was chosen for the ROS assay.

**Figure 5:**
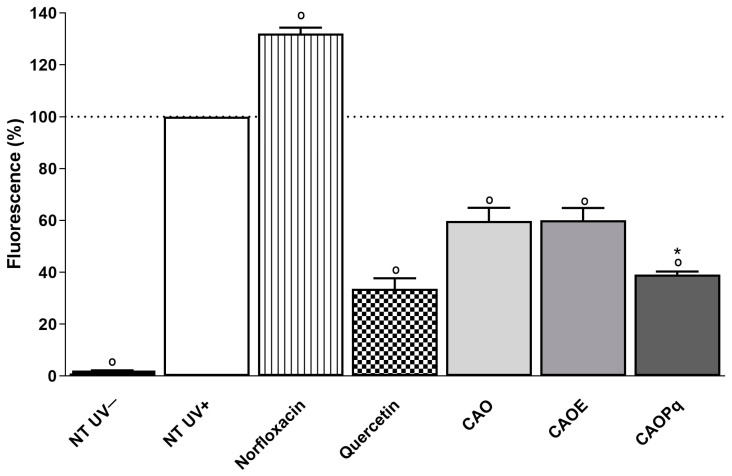
Intracellular reactive oxygen species generation in 3T3 murine fibroblasts after UVA irradiation (4 J/cm^2^) using a fluorescent probe DCFH_2_-DA. The results are expressed as % fluorescence. CAO (AVO + OMC); CAOE (AVO + OMC + EHMCR); CAOPq (AVO + OMC + 5% of PqSE). Concentration C1 (37.02 µg/mL AVO, 74.04 µg/mL OMC and 46.4 µg/mL EHMCR/PqSE). Positive control for ROS generation: Norfloxacin (100 µg/mL). Positive control for antioxidant activity: quercetin (10 µg/mL). Combinations tested: CAO; CAOE; CAOPq. Concentration C1 (37.02 µg/mL AVO, 74.04 µg/mL OMC and 46.4 µg/mL EHMCR/PqSE). The results are expressed as mean ± standard errors of the mean of three independent experiments (*n* = 3). ° Statistically different from irradiated untreated (NT UV+). * No significant difference to quercetin (*p* > 0.05).

The protection against UVA-induced intracellular ROS production was performed using the DCFH_2_-DA probe. The combinations CAO, CAOE, and CAOPq were tested only at concentrations of 37.02 µg/mL of AVO, 74.04 µg/mL of OMC, and 46.4 µg/mL of EHMCR/PqSE. It is possible to observe that when compared to non-treated and irradiated cells (NT + UV), norfloxacin induced a statistically significant increase in ROS generation (32%), and quercetin significantly reduced ROS generation (66%), as expected for pro-oxidant and antioxidant controls, respectively (Figure 5).

CAO and CAOE induced a ROS reduction of 40.3% and 39.9%, respectively, at the tested concentration, whereas the CAOPq induced a higher ROS reduction, reaching 60.9%, when compared to the untreated irradiated cells (NT + UV) (*p* < 0.05). Furthermore, for CAOPq, no significant difference to quercetin was found.

### 3.6. Radical Protection Factor (RPF)

The RPF of the formulations F, FAO, FAOE, and FAOPq (containing 1% and 5% of PqSE) were evaluated by EPR spectroscopy (Table 4). Formulation F, which is a base cream without UV filters, showed a lower RPF (64 ± 6 × 10^14^ radicals/mg) than FAO and FAOE (90 ± 5 and 93 ± 5 × 10^14^ radicals/mg, respectively). When the ingredient EHMCR was replaced by 1% PqSE in formulation FAOPq, the RPF significantly increased compared to all other tested formulations to 857 ± 94 × 10^14^ radicals/mg, and with a concentration of 5% of PqSE, increased even further to 3258 ± 244 × 10^14^ radicals/mg. These results indicate that PqSE possesses a remarkable antioxidant potential, and its antioxidant activity is dependent on the concentration used.

### 3.7. Quantification of Cumulative Radical Production Induced by VIS + NIR Irradiation on Porcine Ear Skin by EPR Spectroscopy

**Figure 6:**
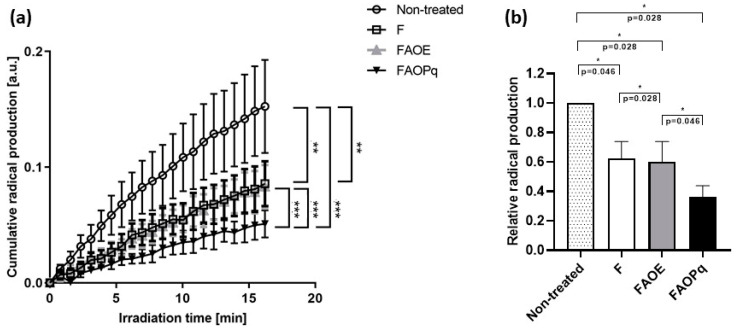
Radical production on porcine ear skin after 15 min of VIS/NIR irradiation. (**a**) Cumulative radical production over 15 min VIS/NIR irradiation, (**b**) Total amount of radicals after 15 minutes of VIS/NIR irradiation in skin; F (Base cream formulation); FAOE (AVO + OMC + EHMCR); FAOPq (AVO + OMC + 5% of PqSE). (*n* = 6 ± SEM). * Statistically significance difference is noted as * (*p* ≤ 0.05), ** (*p* ≤ 0.01) and *** (*p* ≤ 0.001).

Formulations F, FAOE, and FAOPq (containing 1% PqSE) were selected to evaluate their free radical protection effect on porcine ear skin during VIS/NIR irradiation using EPR spectroscopy. The cumulative production of radicals over 15 min of irradiation is demonstrated in Figure 6. Skin treated with F, FAOE, and FAOPq exhibited significant reductions in radical production (*p* < 0.05 and 0.01). Skin treated with formulations F and FAOE had very similar radical reduction (38% and 40%, respectively) when compared with the non-treated skin. FAOPq showed the strongest reduction in cumulative radical production, namely 64% vs. non-treated skin.

### 3.8. Confocal Raman Microspectroscopy (CRM)

**Figure 7:**
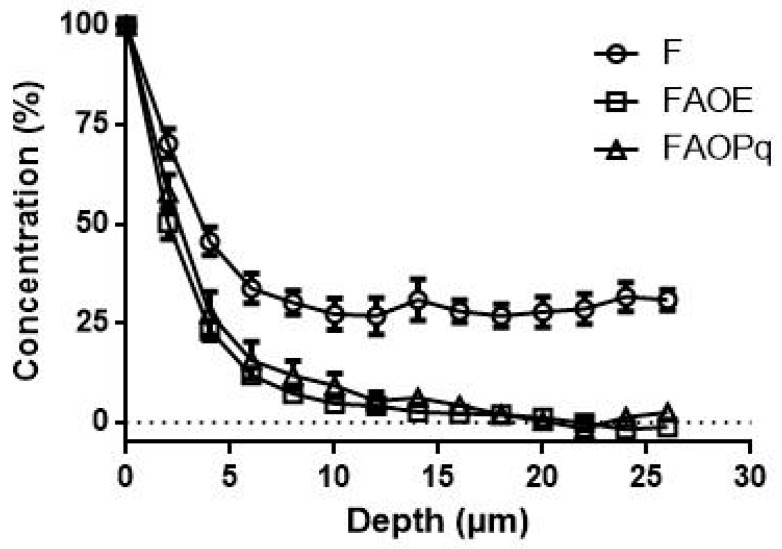
Normalized penetration depth profiles of formulations F, FAOE, and FAOPq into porcine ear skin obtained after 30 min of penetration time using CRM compared to untreated skin. F (Base cream formulation); FAOE (AVO + OMC + EHMCR); FAOPq (AVO + OMC + 5% of PqSE). (*n* = 6). Mean ± SEM.

Formulations F, FAOE, and FAOPq were used for determining the skin penetration properties by confocal Raman microspectroscopy. Figure 7 shows that for formulation F, a contribution can be seen throughout the entire measurement depth of 25 µm. For formulations FAOE and FAOPq, no contribution could be detected below ≈ 17 µm. Also, the amount of penetrated formulation is considerably larger for F. Formulations FAOE and FAOPq showed a very similar penetration behavior into the epidermis.

## 4. Discussion

The daily use of sunscreens is crucial for maintaining healthy skin due to the potential damage induced by UV radiation. However, studies have emerged concerning coral contaminations in the sea caused by the chemical UV filters found in sunscreen formulations [39,40]. Consequently, this study aims to show that a sustainable and natural ingredient such as piquia shells may be used as an ingredient for a sunscreen formulation.

Piquia have been reported as a source of bioactives, rich in phenolic compounds and carotenoids. Additionally, they show low cytotoxicity and possess anti-inflammatory and antioxidant capabilities, as observed through the inhibition of nitric oxide production, and the values obtained are even higher compared to those found in the pulp of the piquia fruit [14,21]. Shells have a higher mass percentage compared to fruit pulp (approximately 60%), and the use of these natural products could add value to what is typically discarded, generating income for the population and contributing to the sustainable development of the Amazon region.

Firstly, a sunscreen formulation must be safe, effective, and without chemical instability. It is known that the combination of AVO and OMC is considered photounstable in formulations, and strategies are necessary to solve this problem, such as a combination with photostabilizers and antioxidant compounds [6,41,42]. When the synthetic photostabilizer EHMCR was associated with AVO and OMC, this combination was considered photostable, as described by Scarpin and coauthors [2]. In this study, we aimed to explore whether the combination of the natural ingredient PqSE with a combination of AVO and OMC could exhibit a similar photostabilizing effect in formulations compared to the synthetic photostabilizer EHMCR. In this study, we developed three different formulations: FAO, which contained AVO and OMC; FAOE, consisting of AVO, OMC, and EHMCR (as described by [2]); and FAOPq, which incorporated AVO, OMC, and PqSE.

Formulation FAO containing AVO and OMC showed a substantial decrease in UVA/UVB ratio after UVA exposure, indicating photoinstability. In the study conducted by Lhiaubet-Vallet et al. [4], it was proposed that the combination of certain components, specifically cinnamate molecules and the enol form of AVO, can undergo various photoreactions. These photoreactions include unimolecular processes such as isomerization and fragmentation, as well as a photocycloaddition reaction known as the De Mayo reaction [43]. The De Mayo reaction is a type of photocycloaddition reaction, specifically [2 + 2] that occurs between two cinnamate molecules or between the enol form of AVO and cinnamate. This reaction leads to the formation of cinnamate dimers or cyclobutylketone photoadducts. However, as a consequence, these photoadducts can further undergo fragmentation, resulting in subsequent fragmentation products.

When EHMCR was combined with these UV filters, the formulation FAOE became photostable [2]. EHMCR is a known photostabilizer and was able to neutralize the photoinstability of retinol, retinyl palmitate, and trans-resveratrol [44,45]. A qualitative fluorescence quenching experiment revealed that retinol, in its excited state, acts as a donor, transferring its excited-state energy to the acceptor, EHMCR. EHMCR then dissipates this energy in a non-destructive manner. This unexpected discovery explains why formulations containing these retinoids and EHMCR exhibit exceptional photostability [44]. A similar experiment with trans-resveratrol showed that singlet energy transfer might play a crucial role in how EHMCR inhibits UV-induced trans-cis isomerization of resveratrol [45]. EHMCR has no phototoxic potential [2] and was also able to stabilize AVO and OMC via singlet–singlet stabilization mechanism [2,46]. Formulations FAOE and FAOPq demonstrated the smallest decrease in the UVA/UVB ratio after UVA exposure. This finding suggests that both EHMCR and PqSE can enhance the photostability of formulations containing AVO and OMC.

Additionally, it was observed that formulation FAOPq, which includes AVO, OMC, and PqSE, exhibited photostability. Furthermore, both FAOPq and FAOE demonstrated higher UV absorption compared to formulation FAO. Rangel and coauthors [33] showed the ability of marine natural products to be used as ingredients for photoprotective formulations. He and collaborators [47] also found that the presence of phenolic compounds in extracts can have several beneficial effects on UV radiation filtration, SPF (Sun Protection Factor), and antioxidant action. However, it was observed that 0.1% of the phenolic antioxidant rosmarinic acid could not stabilize the photounstable combination of the UV filters (AVO + OMC) [48]. Indeed, the result obtained with PqSE in the study represents a significant and novel finding in the literature. The ability of PqSE, a natural and sustainable raw material, to act as a photostabilizing agent in photoprotective formulations opens up new possibilities for the development of sunscreen products.

The phototoxicity results were in line with a previous study [2] since CAO* at proportion 5:7, for AVO and OMC, respectively, was considered phototoxic. Both combination CAOE* and CAOPq* were not considered phototoxic. As discussed before, AVO and OMC photodegradation leads to the production of toxic products and ROS, which can damage fibroblasts decreasing the cell viability after UVA irradiation [2,29,49]. On the other hand, the combination CAOPq* containing AVO, OMC, and PqSE showed no phototoxic potential, which suggests that PqSE reduced the phototoxic potential of CAO*.

Based on further findings [14,21], it is suggested that the lack of phototoxic potential observed in the combination of PqSE could be attributed to the ability of phenolic compounds present in PqSE to neutralize reactive species induced by UVA radiation. Thus, although no phototoxic potential was detected, compatibility, phototoxicity, and photoallergy studies on finish products must be performed in humans since the proposed in vitro test cannot predict the exact incidence of phototoxic reactions in humans.

None of the tested formulations (FAO, FAOE, and FAOPq) showed irritant potential on the HET-CAM assay since they did not induce any damage to the chorioallantoic membrane.

The photoprotective effect against UVB irradiation on keratinocytes (HaCaT) monolayer showed that CAOE was not able to protect keratinocytes against UVB irradiation at all tested concentrations. In contrast, at low concentrations, PqSE presented high protection for UVB irradiation similar to positive control BZP, with cellular viability higher than 80%. The UVB photoprotection effect of PqSE can be explained by the presence of phenolic compounds, specifically gallic and ellagic acids, which are the main constituents of PqSE [21,22]. Roxo et al. also identified a major group of the hydrolyzable tannins (including ellagi- and gallotannins) and triterpenoid saponins as constituents of a hydroalcoholic (8:2 ethanol/water) extract of piquiá shells [50]. PqSE has a maximum absorption peak of around 270 nm, which is within the UVB range. This can be attributed to the hydroxybenzene structures of gallic acid, as suggested by Wang et al. [51]. Additionally, Milani et al. [52] reported that the extract of byproducts from guava fruit (Psidium guajava), which is standardized in ellagic acid, demonstrated a synergistic effect with OMC, leading to an increased SPF related to UVB protection of the formulation compared to the formulation without the extract.

Regarding the protection against ROS induced by UVA radiation on 3T3 fibroblasts monolayer, combination CAOPq induced a higher ROS reduction than CAO and CAOE, reaching 60.9%, when compared to the untreated irradiated cells (NT + UV) (*p* < 0.05). Furthermore, no significant difference between CAOPq and quercetin was found.

Similar results were observed when the ingredients were studied in the formulations FAO, FAOE, and FAOPq by EPR spectroscopy to assess their radical scavenging activity (RPF). EPR is considered the gold standard technique for investigating free radicals directly [35,53,54].

Formulations FAO and FAOE exhibited RPF values of 90 ± 5 and 93 ± 5 × 10^14^ radicals/mg, respectively. This result suggests that the photostabilizer EHMCR did not significantly improve its antioxidant potential compared to FAO. Even the base cream formulation, labeled as F, demonstrated an RPF value of 64 ± 6 × 10^14^ radicals/mg. This antioxidant activity in formulation F can be attributed to the presence of butylhydroxytoluene (BHT) in the formulation. BHT acts as an antioxidant, preventing oxidation of the formulation, and interacts with the DPPH probe through a hydrogen-atom-transfer mechanism, indicating its antioxidant potential [55,56].

FAO contains BHT, along with the UV filters AVO and OMC. Similarly, FAOE includes BHT, AVO, OMC, and EHMCR, an important photostabilizer. However, the addition of EHMCR to FAOE did not significantly improve its antioxidant potential compared to FAO. In contrast, when EHMCR was replaced by PqSE in the FAOPq formulation, a notable concentration-dependent increase in antioxidant potential was observed. FAOPq exhibited RPF values of 857 ± 94 and 3258 ± 244 × 10^1^⁴ radicals/mg for 1% and 5% PqSE concentrations, respectively, demonstrating the strong antioxidant activity of PqSE in the formulation.

The antioxidant activity of a sunscreen formulation containing only UV filters (AVO, OMC, and ethylhexyl dimethyl PABA) was evaluated and compared with a formulation that included 10% grape pomace extract [57]. The researchers found that even the formulation with just UV filters exhibited some antioxidant activity, although at a lower level. However, when the grape pomace extract was added, the antioxidant potential of the formulation was tripled. Your observation regarding the similarities in results between FAO, FAOE, and FAOPq, with higher antioxidant activity when PqSE was used as an ingredient, aligns with the findings of the previous study [57]. This suggests that PqSE, with its phenolic compounds and antioxidant properties, contributes significantly to the enhanced antioxidant activity observed in these formulations.

The investigations of the cumulative radical production after VIS/NIR irradiation on porcine ear skin ex vivo by EPR spectroscopy demonstrated for all testes cream formulations (F, FAOE, and FAOPq (containing 1% of PqSE)) a significant reduction in radical production vs. non-treated skin. Formulations F and FAOE reduced 38% and 40%, respectively, of radical production on porcine ear skin, while FAOPq decreased 64% of cumulative radical production. A main cause for this difference in the antioxidant potential among formulations is the presence of the natural compound PqSE, a hydroalcoholic extract rich in polyphenol compounds, such as gallic and ellagic acids, which are well-known antioxidant agents. Freitas and co-authors [26] highlighted the benefits when the polyphenol trans-resveratrol was combined with beta-carotene and UV filters (OMC, AVO, octocrylene, and bemotrizinol) since the formulation showed better safety and efficacy. Stevanato and co-authors [58] described an antioxidant potential and protection against UV-induced ROS by 14 natural polyphenols.

The penetration depth of formulations, which was evaluated using Raman confocal microspectroscopy into porcine ear skin, showed that the base cream without UV filters (Formulation F) that contains several emollient agents allowed a greater penetration into porcine ear skin. These results are in line with the ones obtained by Zhang et al. [59], who observed that both a placebo cream and cream-rich antioxidants could permeate the stratum corneum and might reach the metabolically active layers of the viable epidermis. However, to prove this experimentally, further investigations are necessary. Infante et al. [38] also mentioned that the porcine ear skin is an appropriate skin model for penetration studies into stratum corneum (SC) but may be more permeable than human SC. In the case of FAOE and FAOPq, the study found that they exhibited around 5% penetration into the SC, reaching depths of 14 µm and 16 µm, respectively. This suggests that they did not penetrate deeply into the viable epidermis but remained predominantly within the SC.

This study aims to identify a new ingredient for sunscreen formulations. It is well-established that UV filters should remain on the surface of the skin, specifically in the stratum corneum (SC), to effectively reflect, absorb, or neutralize UV radiation and prevent damage [60]. Penetration of UV filters into deeper layers should be avoided, as it can lead to adverse effects such as an increased risk of phototoxicity, systemic toxicity, or photoallergic reactions [42,61]. Recent studies have shown systemic absorption of oxybenzone (benzophenone-3), with the chemical being detected in human blood plasma, urine, amniotic fluid, and breast milk following skin application. Additionally, oxybenzone poses a threat to coral reef conservation, compromising their resilience to climate change [62]. Research has demonstrated that exposure to oxybenzone results in increased DNA damage and deformities in coral species such as *Stylophora pistillata*, as well as changes in the metabolomic profiles of *Pocillopora damicornis* [63]. As the public’s use of sunscreens rises, the FDA proposes that more data are needed, leading to more safety information for several common UV filters [64].

In the study, PqSE demonstrated significant antioxidant potential when evaluated through in chemico tests, in vitro studies, and ex vivo assessments. It exhibited the ability to protect against UVA, UVB, and VIS/NIR irradiation by reducing the generation of reactive oxygen species (ROS) in all the conducted tests.

These findings highlight PqSE as a promising ingredient that can provide broad-spectrum protection against different types of radiation while minimizing ROS production. However, further research is necessary to confirm these observations, ensuring the safety and efficacy of PqSE in various skin types and real-world conditions.

Nicolai et al. [65] observed that *P. ecklonii* extract in an ethanol/propylene glycol solution did not permeate through the skin and suggested that its antioxidant and free radical scavenging activity could primarily occur on the skin’s surface. Considering the results from both studies, FAOPq in the current study and the *P. ecklonii* extract from Nicolai et al. 2020 [65] study, it appears that these ingredients exhibit their antioxidant potential and free radical scavenging activity primarily on the skin’s surface rather than penetrating into deeper skin layers. This information further supports the potential of PqSE as an ingredient for sunscreen formulations because it suggests that its antioxidant effects can be exerted directly on the skin’s surface, providing protection against free radicals and oxidative damage. Nevertheless, additional research is needed to confirm these findings and explore the potential benefits of PqSE in practical sunscreen applications.

## 5. Conclusions

In this study, we highlighted the hydroalcoholic extract obtained from shells of the Amazonian fruit piquia (*Caryocar villosum*), considered a byproduct of piquia fruit since its shells are frequently discarded, as a natural and sustainable ingredient for a sunscreen formulation. The study showed that piquia shell extract (PqSE), when in formulation, was able to stabilize a photounstable combination of UV filters, avobenzone (AVO), and octyl methoxycinnamate (OMC), and it could also decrease the phototoxicity of these UV filters. PqSE exhibited strong antioxidant properties in chemico tests, in vitro studies, and ex vivo evaluations. It successfully mitigated the production of reactive oxygen species (ROS) in response to UVA, UVB, and VIS/NIR radiation, demonstrating its protective potential across all test conditions. Moreover, the formulation with PqSE did not present irritant potential and did not penetrate into the viable epidermis of porcine ear skin, being able to exert its photoprotective and antioxidant potential on the stratum corneum of the skin.

Based on the results obtained from the various tests conducted in this study, PqSE exhibited superior performance compared to the well-known synthetic photostabilizer ethylhexyl methoxycrylene (EHMCR). The study concluded that PqSE could potentially be an alternative to EHMCR in sunscreen formulations due to its improved results in terms of photosafety and efficacy.

Furthermore, the combination of PqSE with UV filters presents an opportunity to develop a sunscreen formulation using a natural and sustainable ingredient. These findings highlight the possibility of utilizing fruit byproducts as an interesting strategy, allowing for the reuse of these residues while maintaining good results in in vitro/ex vivo safety and efficacy evaluations. Moreover, they can serve as sustainable raw materials for the development of sunscreens, ensuring not only effectiveness but also environmental sustainability.

## Figures and Tables

**Table 1:** Composition of the different formulations F, FAO (with AVO + OMC), FAOE (with AVO + OMC + EHMCR), and FAOPq (AVO + OMC + PqSE) (% *w*/*w*) submitted to photostability, HET-CAM, RPF, radical production by EPR and confocal Raman microspectroscopy investigations. * Safety tests (HET-CAM, Photostability) were performed with 5% of piquia extract. Efficacy tests (EPR, confocal Raman microspectroscopy) were performed with 1% of piquia extract.

Ingredients	F	FAO	FAOE	FAOPq
Cetearyl Alcohol and Cetearyl Glucoside	3.0	3.0	3.0	3.0
C12-15 Alkyl Benzoate	5.0	5.0	5.0	5.0
Isopropyl myristate	3.0	3.0	3.0	3.0
butylated hydroxytoluene	0.05	0.05	0.05	0.05
Glycerol	2.0	2.0	2.0	2.0
Hydroxyethyl Acrylate/Sodium acryloyldimethyl taurate copolymer and squalane and polysorbate 60	1.0	1.0	1.0	1.0
Propylene glycol	2.0	2.0	2.0	2.0
Phenoxyethanol and parabens	0.8	0.8	0.8	0.8
Cyclopentasiloxane	2.0	2.0	2.0	2.0
Avobenzone (AVO)	-	4.0	4.0	4.0
Octyl methoxycinnamate (OMC)	-	8.0	8.0	8.0
Ethylhexyl methoxycrylene (EHMCR)	-	-	5.0	-
Piquia shells hydroalcoholic extract (PqSE)	-	-	-	1.0 or 5.0 *
Water	81.15	69.15	64.15	64.15 or 68.15

**Table 2:** Results of the phototoxicity test (3T3 NRU PT) for UV filter combination. Positive control: norfloxacin. Combinations tested: CAO (AVO + OMC), CAO* (AVO + OMC), CAOE* (AVO + OMC + EHMCR), and CAOPq* (AVO + OMC+ 5% of PqSE). The results are expressed as a mean photoeffect (MPE) of two independent experiments (*n* = 2). * Indicates the phototoxic proportion 5:7 for AVO and OMC, respectively.

Samples	MPE	IC_50_ (−UV) (µg/mL)	Probability of Toxicity
Norfloxacin	0.501 0.434	- -	Phototoxic/ noncytotoxic
CAO (4:8)	0.035 0.026	20.373 12.36	Nonphototoxic/cytotoxic
CAO* (5:7)	0.233 0.269	36.714 -	Phototoxic/ cytotoxic
CAOE* (5:7:5)	−0.046 0.007	15.532 23.982	Nonphototoxic/cytotoxic
CAOPq* (5:7:5)	0.005 0.073	14.254 69.307	Nonphototoxic/cytotoxic

**Table 3:** Mean and standard deviation (SEM) of ocular irritation scores and classification of tested samples by the HET-CAM test. NaCl 0.9% and SDS 1% were used as negative and positive controls for irritation, respectively. (*n* = 4). Formulations tested: FAO (AVO + OMC); FAOE (AVO + OMC+ EHMCR); FAOPq (AVO + OMC + 5% of PqSE).

Samples	Irritation Score	Classification
NaCl 0.9%	0.0 ± 0.0	Non-irritating
SDS 1%	12.0 ± 0.0	Irritating
FAO	0.0 ± 0.0	Non-irritating
FAOE	0.0 ± 0.0	Non-irritating
FAOPq	0.0 ± 0.0	Non-irritating

**Table 4:** Radical protection factor (RPF) of formulations. F (Base cream formulation); FAO (AVO + OMC); FAOE (AVO + OMC + EHMCR); FAOPq 1% (AVO + OMC + 1% of PqSE); FAOPq 5% (AVO + OMC + 5% of PqSE).

Formulation	RPF (× 10^14^ radicals/mg)
F	64 ± 6
FAO	90 ± 5
FAOE	93 ± 5
FAOPq 1%	857 ± 94
FAOPq 5%	3258 ± 244

## Data Availability

The data is available on request from the authors.
